# The Density of Perineuronal Nets Increases With Age in the Inferior Colliculus in the Fischer Brown Norway Rat

**DOI:** 10.3389/fnagi.2020.00027

**Published:** 2020-02-11

**Authors:** Amir M. Mafi, Lindsay N. Hofer, Matthew G. Russ, Jesse W. Young, Jeffrey G. Mellott

**Affiliations:** Department of Anatomy and Neurobiology, Northeast Ohio Medical University, Rootstown, OH, United States

**Keywords:** inferior colliculus, perineuronal nets, *Wisteria floribunda* agglutinin, GABAergic, aging

## Abstract

Age-related hearing loss, one of the most frequently diagnosed disabilities in industrialized countries, may result from declining levels of GABA in the aging inferior colliculus (IC). However, the mechanisms of aging and subsequent disruptions of temporal processing in elderly hearing abilities are still being investigated. Perineuronal nets (PNs) are a specialized form of the extracellular matrix and have been linked to GABAergic neurotransmission and to the regulation of structural and synaptic plasticity. We sought to determine whether the density of PNs in the IC changes with age. We combined *Wisteria floribunda* agglutinin (WFA) staining with immunohistochemistry to glutamic acid decarboxylase in three age groups of Fischer Brown Norway (FBN) rats. The density of PNs on GABAergic and non-GABAergic cells in the three major subdivisions of the IC was quantified. Results first demonstrate that the density of PNs in the FBN IC increase with age. The greatest increases of PN density from young to old age occurred in the central IC (67% increase) and dorsal IC (117% increase). Second, in the young IC, PNs surround non-GABAergic and GABAergic cells with the majority of PNs surrounding the former. The increase of PNs with age in the IC occurred on both non-GABAergic and GABAergic populations. The average density of PN-surrounded non-GABAergic cells increased from 84.9 PNs/mm^2^ in the young to 134.2 PNs/mm^2^ in the old. While the density of PN-surrounded GABAergic cells increased from 26 PNs/mm^2^ in the young to 40.6 PNs/mm^2^ in the old. The causality is unclear, but increases in PN density in old age may play a role in altered auditory processing in the elderly, or may lead to further changes in IC plasticity.

## Introduction

The extracellular matrix in the central nervous system, which represents 10–20% of brain volume, can form lattice-like structures known as perineuronal nets (PNs) that surround somas and apical dendrites of neural subpopulations (Cragg, 1979; Karetko and Skangiel-Kramska, 2009; Sonntag et al., 2015). PNs can surround a variety of types of neurons throughout the brain although most reports focus on their relationship to GABAergic cells (Kosaka and Heizmann, 1989; Härtig et al., 1992; Sonntag et al., 2015). PNs and their primary molecular component, chondroitin sulfate proteoglycans (CSPGs), are critical for numerous functions including ion buffering, stabilizing high-rate synaptic transmission, modulating cellular integrity, neuroprotection, mechanical stabilization of synaptic contacts, and inhibition of structural plasticity (see reviews Sonntag et al., 2015; Bosiacki et al., 2019; Testa et al., 2019). Of these functions the inhibition of plasticity has garnered the most attention as the enzymatic disruption of PNs can restore juvenile-like plasticity in the adult cortex (Pizzorusso et al., 2002, 2006; Gogolla et al., 2009). This apparent role for PNs in the diminished neural plasticity that characterizes the aging brain, along with an increasing aging population, has generated strong interest in the role of PNs in the cortex during neurodegenerative diseases and aging (see reviews Burke and Barnes, 2006; Karetko and Skangiel-Kramska, 2009; Bosiacki et al., 2019; Duncan et al., 2019; Testa et al., 2019).

Many studies of PN expression investigate structural plasticity in cortex in neurological disorders (e.g., Alzheimer’s, autism, epilepsy, schizophrenia, etc.) and traumatic brain injury (Soleman et al., 2013; see reviews: Bosiacki et al., 2019; Duncan et al., 2019; Testa et al., 2019). Studies examining the role of PNs in physiological aging are less common and largely address questions in cortex regarding memory and cognition (Foscarin et al., 2017). Furthermore, the role of PNs in aging sensory systems has not been broadly studied and are also commonly restricted to cortex (Karetko-Sysa et al., 2014). Studies that have been conducted in sensory cortices are generally in agreement that PNs increase with age (Tanaka and Mizoguchi, 2009; Yamada and Jinno, 2013; Karetko-Sysa et al., 2014; Ueno et al., 2018). However, reports in the aging auditory cortex have been inconsistent: Ueno et al. (2018) found an overall increase of PNs in the auditory cortex, Brewton et al. (2016) found a decrease of PNs, and Cisneros-Franco et al. (2018) found an increase of PNs early in life followed by a loss during old age. Even though PNs are found throughout the auditory system (Sonntag et al., 2015), the effects of aging on these subcortical populations have not been well studied.

The inferior colliculus (IC) is a large midbrain nucleus that processes most auditory inputs and integrates temporal and spectral properties before signals reach the thalamus and cortex. The IC may be an ideal model to examine the functions of PNs with age. First, there is a sizeable population of PNs in the functionally distinct IC subdivisions (central IC, ICc; dorsal cortex IC, ICd; lateral cortex IC, IClc) that surround both GABAergic and glutamatergic cells (Foster et al., 2014; Fader et al., 2016; Beebe and Schofield, 2018). Second, there are well-known age-related changes to GABAergic neurotransmission in the IC that include the downregulation of GABA synthesis, postsynaptic changes to the subunit composition of GABA_A_ receptors, and the loss of GABAergic synapses onto both GABAergic and glutamatergic neurons (see reviews: Caspary et al., 2008; Syka, 2010; Caspary and Llano, 2018). Third, the IC can undergo age-related tonotopic plasticity when there is a loss of high frequency input from the cochlea (Willott, 1986; Frisina and Rajan, 2005). Lastly, changes to GABAergic neurotransmission in the IC can lead to a variety of temporal processing deficits (Palombi and Caspary, 1996a; Walton et al., 1998, 2002; Frisina, 2001; Parthasarathy and Bartlett, 2011; Pollak et al., 2011; Cai et al., 2014). The altered presence of PNs at specific ages, and the potential subsequent changes in structural and synaptic plasticity, may have substantial functional consequences for the processing of acoustic information at subcortical levels.

In the current study, we combined *Wisteria floribunda* agglutinin (WFA) staining with antibodies to glutamic acid decarboxylase (GAD) to distinguish GABAergic and non-GABAergic (i.e., glutamatergic; Ito et al., 2011; Ito and Oliver, 2012) neurons that were surrounded by PNs in the Fischer Brown Norway (FBN) rat. Our objective was to determine (1) whether the density of PNs in the IC subdivisions changed with age and (2) whether changes were specific to either GABAergic or non-GABAergic IC cell populations. The FBN strain is considered a superior model for aging by the National Institute on Aging due to its longer median life span relative to other rat strains and mice, and FBN rats have been used in numerous studies investigating the age-related changes of inhibitory neurotransmission in the peripheral and central auditory systems (Milbrandt and Caspary, 1995; Ling et al., 2005; Turner et al., 2005; Caspary et al., 2008; Wang et al., 2009; Hughes et al., 2010; Richardson et al., 2011, 2013; Cai et al., 2018). In a previous studies, we separated FBN rats into four age groups, as there are significant age-related changes to GABA_A_R subunit composition as early as 4 months (Robinson et al., 2019). In the current study we use three age groups as we did not find that PN density was significantly different within our 2–7 months group. Accordingly, we demonstrate that PNs surround both non-GABAergic and GABAergic IC cells in FBN rats. Regardless of age, PNs were more commonly surrounding non-GABAergic IC cells in each age group examined. We also find that with mixed-effect modeling: (1) the density of PNs increased in the oldest age group (28–29 months) on both non-GABAergic and GABAergic IC cells and (2) when subdivisions were considered, the density of PNs increased in the oldest age group on non-GABAergic in each subdivision.

## Materials and Methods

### Animals

All procedures were conducted in accordance with the Northeast Ohio Medical University Institutional Animal Care and Use Committee and NIH guidelines. Results are described from 15 male FBN rats (National Institute of Aging, Bethesda, MD, United States) weighing 185–622 g across three age groups: 2–7 months; 19–24 months; 28–29 months. Efforts were made to minimize the number of animals and their suffering.

### Perfusion and Tissue Processing

Each animal was deeply anesthetized with isoflurane and perfused transcardially with 0.9% saline solution, followed by 250 ml of 4% paraformaldehyde in 0.1M phosphate buffer, pH 7.4 and then by 250 ml of the same fixative with 10% sucrose. The brain was removed and stored at 4°C in fixative with 25–30% sucrose for cryoprotection. The following day the brain was prepared for processing by removing the cerebellum and cortex and blocking the remaining piece with transverse cuts posterior to the cochlear nucleus and anterior to the thalamus. The tissue was frozen and cut on a sliding microtome into 40 μm thick transverse sections that were collected in six series.

Putative GABAergic cells were stained with immunochemistry for GAD. The GAD antibody used here has been validated in a previous study through immunoblotting analysis and absorption tests with fusion proteins (Ito et al., 2007). The sections were pretreated with 0.2% Triton X-100 for permeabilization and 3% bovine serum albumin/1.5% normal goat serum to limit non-specific labeling. Sections were incubated overnight for 1–2 days at 4°C with mouse anti-GAD monoclonal antibody (GAD67; #MAB5406 Millipore, RRID:AB_2278725, diluted 1:400). Next, sections were incubated for 1 h at room temperature with an Alexa Fluor 647-tagged goat anti-mouse secondary antibody (to reveal GAD67; diluted to 1:100; #ab150115 Life Technologies). Tissue was then washed in phosphate-buffered saline (PBS) and then stained with fluorescein-labeled Wisteria Floribunda Lectin (WFA; 1:100; Vector Laboratories) and then tagged with another AlexaFluor in near infrared (AF750) to stain PNs. Near infrared markers (AF647 and AF750) were chosen to help minimize autofluorescent signals from lipofuscin (a pigment that accumulates in the cytoplasm during aging) and is prominent in standard red and green fluorescent channels (Robinson et al., 2019). Lastly, sections were washed in PBS and incubated for 20 min in a green (500/525) NT (#N-21480 Molecular Probes diluted to 1:100) to label cell bodies in the IC. Sections were mounted on gelatin-coated slides, allowed to dry and coverslipped with DPX (Sigma).

### Data Analysis

Sections were chosen through the mid-rostrocaudal region (between interaural levels 0.12 mm – 0.48 mm; Paxinos and Watson, 1998) of the IC in which the three major IC subdivisions [central IC (ICc), lateral cortex of the IC (IClc) and dorsal cortex of the IC (ICd) are present]. Subdivisions of the IC were delineated according to a rat anatomical atlas of the brain (Paxinos and Watson, 1998) and by their pattern of Nissl staining. Staining revealed PNs surrounding GAD-immunoreactive (GAD^+^) cells and GABA-immunonegative (GAD^–^) cells in each of the IC subdivisions. A cell was identified as either having a PN (PN-surrounded) or not having a PN (non-PN-surrounded). In rare cases a PN was present without an accompanying cell. Immunopositive cells were labeled intensely making it straightforward to distinguish PN-surrounded GAD^+^ and GAD^–^ cells.

Fifteen animals (5 per age group) with robust immunostaining were chosen for quantification. One transverse mid-rostrocaudal IC sections, containing the left and right IC, was quantified per experiment. Every PN in each IC subdivision was quantified. Each PN, GAD^+^ and GAD^–^ cell was manually plotted with a 63x oil-immersion objective (numerical aperture 1.4) aligned to a Neurolucida reconstruction system (MBF Bioscience, Williston, VT, United States) attached to a Zeiss AxioImager M2. Each combination (PN surrounding a GAD^+^ cell, PN surrounding a GAD^–^ cell and, PN without a visible cell) was plotted with a unique marker. NeuroExplorer (MBF Bioscience, Williston, VT, United States) was used to determine the densities of PNs and GAD^+^ cells across the reconstructed IC section. All photomicrographs presented here are 2 μm maximum image projections and were captured with a Zeiss AxioImager M2 fluorescence microscope and Hamamatsu Orca Flash 4.0 camera (Hamamatsu) and optically sectioned at 0.1 μm steps with an Apotome 2 (Zeiss). Adobe Photoshop (Adobe Systems) was used to add scale bars, crop images, erase background around tissue sections, adjust intensity levels and colorize monochrome images. The results of these plots were used for a quantitative summary of the distribution of PNs surrounding GAD^+^ and GAD^–^ cells. Final images of the plots were refined with Adobe Illustrator (Adobe Systems, Inc., San Jose, CA, United States) for preparation of figures.

The GAD immunostaining often failed to penetrate the full thickness of the section, leaving a central slice of the section unstained. By analyzing the data with a 63X objective (NA = 1.4), it was possible to restrict the quantitative analyses to parts of the section that had successful immunostaining (Mellott et al., 2014b). Sections cut at 40–50 μm typically shrink during dehydration such that the final thickness on slides is 20–30 μm. The GAD and WFA staining in these sections usually extended 5–10 and 4–5 μm from each surface, respectively. Thus, a central layer up to 22 μm thick could be left unpenetrated by both antibodies. By paying special attention to focusing on the center of the soma when plotting the symbol for a particular cell, data points were obtained with sufficient resolution in the z plane (section depth) to allow subsequent filtering of the data by depth. This yielded two zones of data from each section (one associated with each surface) in which tissue was well stained with both antibodies, and a central zone that was not well stained. After the data were plotted, the X, Y, and Z coordinates of all markers were exported from Neurolucida to Microsoft Excel and sorted based on the Z coordinate. Markers in the poorly stained central layer were excluded from further analyses. Such depth filtering necessarily reduces the sample size for quantitative comparisons, but a sufficient number of sections were analyzed to yield a substantial number of PNs; 25,491 PNs (2–7 months, 6,896 PNs; 19–24 months, 8,453 PNs; 28–29 months, 10,142 PNs) across the three age groups were analyzed and coded as GAD^+^ or GAD^–^.

Variation in PN density according to age group, IC subdivision, and (where relevant) GAD immunostaining profile was analyzed using linear mixed-effects models. Mixed-effects models allow for a hybrid of repeated measures analysis (i.e., “within-subject” variables), Model I ANOVA fixed factor analysis (i.e., “between-subject” variables), and Model II ANOVA random factor analysis (i.e., variance components), in the same gestalt statistical test. In this study, age group was specified as a between-subjects fixed factor across individual rats, individual IC subdivision and GAD profile were specified as within-subjects fixed factors within individual rats, and individual rat number was specified as a random factor. P-values for pairwise *post hoc* tests of differences between consecutive age groups (i.e., 2–7 months *versus* 19–24 months, 19–24 months *versus* 28–29 months) were adjusted using the False Discovery Rate procedure (Benjamini and Hochberg, 1995), a method that simultaneously limits experiment-wise alpha inflation and minimizes the correlated loss of statistical power. Given the small sample sizes and resultant deviations from normality, raw densities were rank transformed prior to analysis, such that the smallest value (regardless of age group) was given a rank of 1 and the largest value (regardless of age group) a rank of N (where N is the sample size), with intermediate values ranked in sequential order accordingly. This procedure effectively converted our statistical analyses into non-parametric tests, controlling for deviations from normality (Conover and Iman, 1981). All statistical tests were performed in R (version 3.6.0 for Mac OS X; R Core Team, 2019), supplemented by the add-on packages *nlme* (Pinheiro et al., 2019) and *emmeans* (Lenth, 2019).

## Results

We combined staining for PNs, immunolabeling for GAD and fluorescent Nissl to identify PNs that surround GAD^+^ and GAD^–^ cells with age in the three major subdivisions of the IC. We first describe the changes in PN density in the IC and its subdivisions during aging. We then describe the relationship of PNs with GAD^+^ and GAD^–^ cells during aging. Results are described from 25,491 PNs (6,532 PNs surrounded GAD^+^ cells and 18,959 surrounded GAD^–^ cells) across 15 cases, 5 per age group (Table 1).

**TABLE 1:** Summary of cases, ages and the average densities of PNs across the subdivisions of the IC.

Case	Age (months)	Sex	ICc ave. PN/mm^2	ICd ave. PN/mm^2	IClc ave. PN/mm^2	IC ave. PN/mm^2
R39	2	M	77	64	85.4	75
R40	2	M	113.1	73.1	81.5	94
R26	3	M	150.9	129.8	141.2	143
R72	3	M	125.8	74	109	108
R9	5	M	121.9	89.1	104.9	107
R22	19	M	127.4	76	113.7	109
R2	22	M	108.6	116.1	127.5	116
R4	22	M	154.3	91.1	130.1	131
R8	24	M	155.9	152.4	149.6	153
R10	24	M	145.3	162.9	165.2	154
R30	28	M	197.9	176.3	147.2	172
R34	28	M	203.4	171.5	145.8	175
R38	28	M	203.5	205	175.7	197
R78	28	M	178.2	161.6	139.5	164
R36	29	M	205.2	218.8	153.9	181

### Density of PNs in the IC Increases With Age

PNs were readily identified in each IC subdivision of each age group. As described in “Materials and Methods,” the quantitative analysis was restricted to tissue depths that were successfully penetrated by the WFA staining and by the GAD antibody.

**FIGURE 1:**
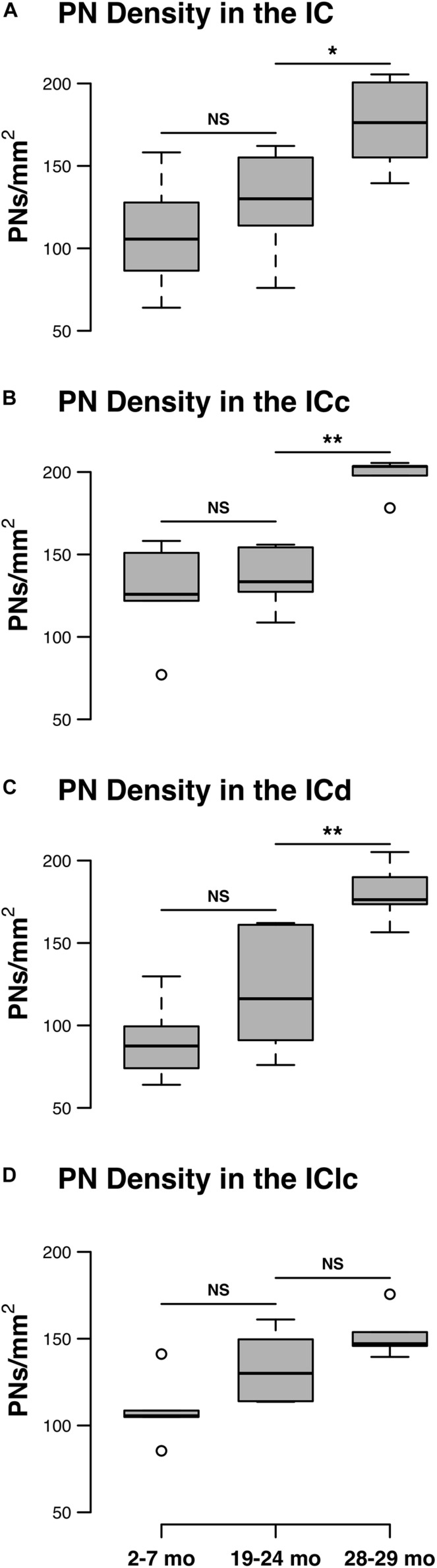
Box plots summarizing the densities (PNs/mm^2^) of PNs in the IC across three age groups. **(A)** Densities of PNs across the IC. Pairwise differences in PN density demonstrated a significant, increase between the 19–24 and 28–29 months age groups (*p = 0.011). **(B)** Densities of PNs in the ICc. Density of PNs was significantly higher at 28–29 months of age compared to the preceding 19–24 months of age group (**p = 0.005). **(C)** Densities of PNs in the ICd. Density of PNs was significantly higher at 28–29 months of age compared to the preceding 19–24 months of age group (**p = 0.007). **(D)** Densities of PNs in the IClc. While there was a trend for the density of PNs to increase with age, densities at 28–29 months of age were not significantly increased as compared to another age (*p* ≥ 0.177). In each box plot, dark lines represent the median of the distribution, boxes extend across the interquartile range, and whiskers extend to ±150% of the interquartile range. Circles indicate outliers beyond this range. See list of abbreviations.

The density of PNs in the IC at 28–29 months of age was 62.2% greater than the density at 2–7 months (Figure 1A). PN density significantly increased between the 19–24 and 28–29 months age groups (*p* = 0.011), but were similar between two younger age groups (i.e., 2–7 months *versus* 19–24 months: *p* = 0.098). There was a significant interaction between age group and IC subdivision, indicating divergent age-related trends in the ICc, ICd, and IClc (*p* = 0.015). We detail trends in individual subdivisions below.

#### Central Nucleus of the IC

**FIGURE 2:**
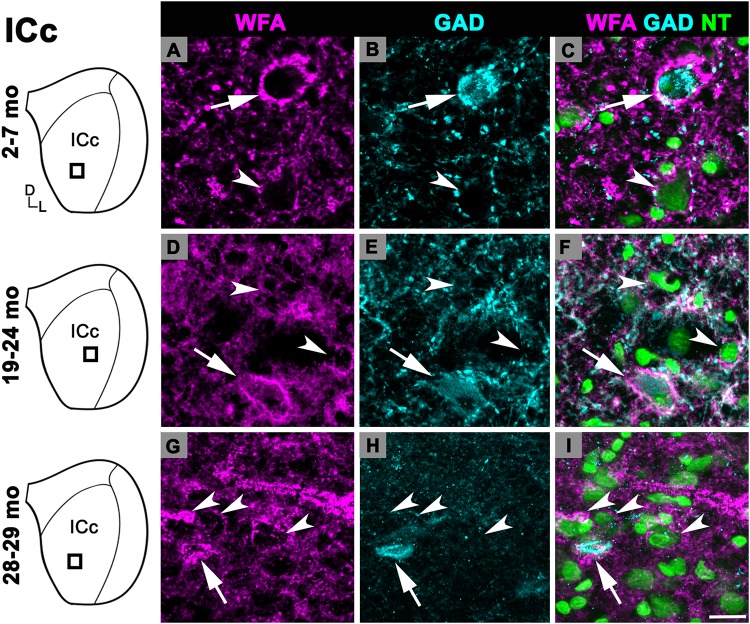
Structured illumination fluorescence images, taken at 0.2 μm steps, showing PNs surrounding GAD^+^ (arrows) and GAD^–^ (arrowheads) cells in the ICc. Boxes in IC schematics indicates the region the images were taken. PNs are shown in magenta, GAD is shown in cyan, and NT is shown in green. Each row represents the ICc across a single age group. **(A–C)** Photomicrographs of a 3 months old ICc. Case R26. **(D-F)** Photomicrographs of a 22 months old ICc. Case R4. **(G–I)** Photomicrographs of 28 months old ICc. Case R30. D, dorsal; L, lateral. See list of abbreviations. Scale bar = 20 μm.

Changes in the density of PNs in the ICc closely resembled the observed changes that were quantified for the entire IC (compare Figures 1A,B). In each age group, PNs surrounded a subpopulation of GAD^+^ and GAD^–^ cells in the ICc (Figure 2; GAD^+^, arrows; GAD^–^, arrowheads). PNs that surround GAD^+^ cells were often thicker than PNs that surround GAD^–^ cells (Figures 2C,F). GABAergic cells, especially larger cells often had a PN (Figures 2C,F). PNs were commonly associated with dense perisomatic rings of GAD^+^ puncta (Figures 2A–C). PN-surrounded GAD^–^ cells varied in size (compare Figures 2C,F,I). The density of PNs at 28–29 months of age significantly increased from the preceding 19–24 age group (*p* = 0.005). In contrast, PN density was statistically similar between the two younger age groups (*p* = 0.606; Figure 1B).

#### Dorsal Cortex of the IC

**FIGURE 3:**
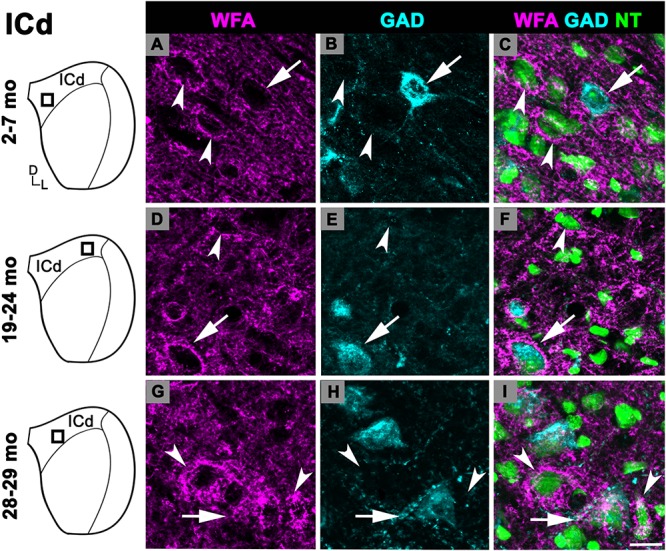
Structured illumination fluorescence images, taken at 0.2 μm steps, showing PNs surrounding GAD^+^ (arrows) and GAD^–^ (arrowheads) cells in the ICd. Boxes in IC schematics indicates the region the images were taken. PNs are shown in magenta, GAD is shown in cyan, and NT is shown in green. Each row represents the ICd across a single age group. **(A–C)** Photomicrographs of a 2 months old ICd. Case R39. **(D–F)** Photomicrographs of 24 months old ICd. Case R10. **(G–I)** Photomicrographs of 28 months old ICd. Case R38. D, dorsal; L, lateral. See list of abbreviations. Scale bar = 20 μm.

There was an age-related increase in the density of PNs throughout the ICd (Figure 1C). PN density at 28–29 months significantly increased relative to the preceding 19–24 months age group (*p* = 0.007). Similarly, there was a nearly significant increase between the 2–7 and 19–24 months age groups (*p* = 0.052). In each age group, PNs surrounded a subpopulation of GAD^+^ and GAD^–^ cells in the ICd (Figure 3; GAD^+^, arrows; GAD^–^, arrowheads). GABAergic cells, especially larger cells often had a PN (Figures 3C,F,I). PN-surrounded GAD^–^ cells varied in size (compare Figures 3C,F,I).

#### Lateral Cortex of the IC

**FIGURE 4:**
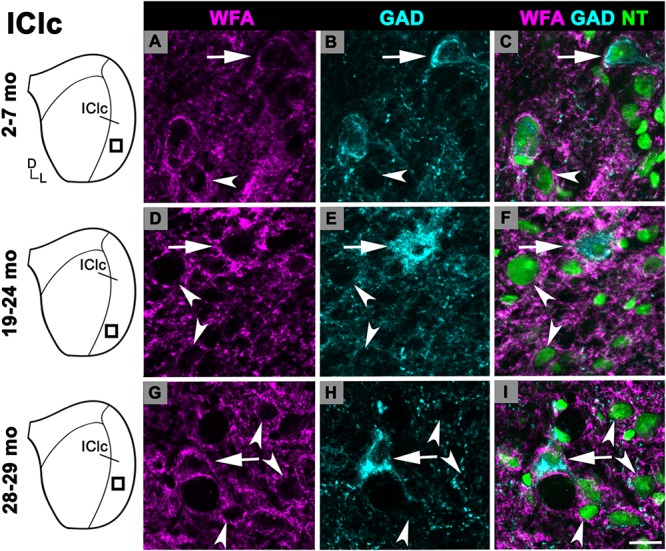
Structured illumination fluorescence images, taken at 0.2 μm steps, showing PNs surrounding GAD^+^ (arrows) and GAD^–^ (arrowheads) cells in the IClc. Boxes in IC schematics indicates the region the images were taken. PNs are shown in magenta, GAD is shown in cyan, and NT is shown in green. Each row represents the IClc across a single age group. **(A–C)** Photomicrographs of a 3 months old IClc. Case R26. **(D–F)** Photomicrographs of a 22 months old IClc. Case R4. **(G–I)** Photomicrographs of 29 months old IClc. Case R36. D, dorsal; L, lateral. See list of abbreviations. Scale bar = 20 μm.

While there was an upward trend (Figure 1D), the density of PNs in the IClc did not significantly increase with age (*p* ≥ 0.177 for all sequential pairwise differences). As in the ICc and ICd, PNs in the IClc surrounded both GAD^+^ and GAD^–^ cells (Figure 4). GABAergic cells in the IClc often had a PN (Figure 4). PN-surrounded GAD^–^ cells varied in size (compare Figures 2C,F,I). of Overall, the density of PNs in the young IClc was similar to the young ICc and ICd. However, the increase in density with age in the IClc was not significant relative to the other subdivisions.

### PNs Surrounding GABAergic and Glutamatergic IC Cells

**FIGURE 5:**
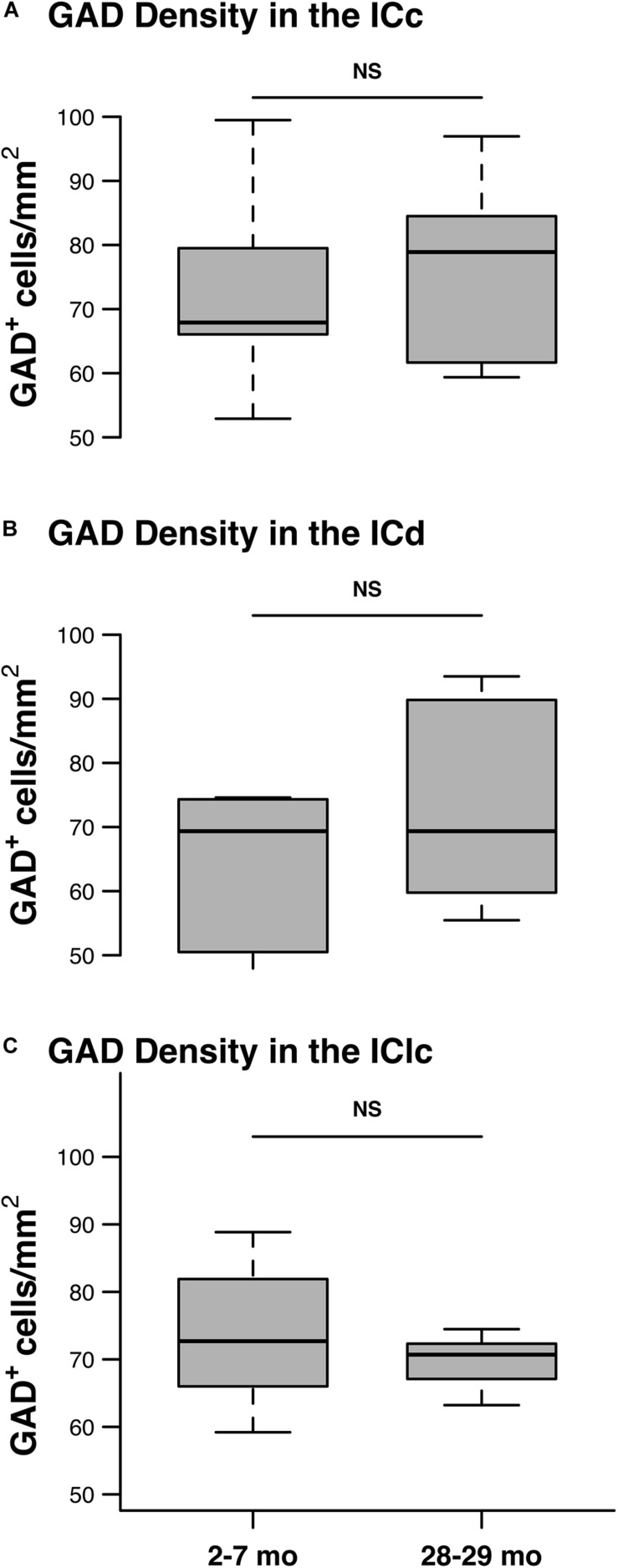
The density of GAD^+^ cells does not significantly change with age. Box plots summarizing the density of GAD^+^ in the youngest and oldest age groups in each IC subdivision. **(A)** The density of GAD^+^ cells was not significantly different between the 2–3 and the 28–29 months old ICc (*p* = 0.53). **(B)** The density of GAD^+^ cells was not significantly different between the 2–3 and the 28–29 months old ICd (*p* = 0.462). **(C)** The density of GAD^+^ cells was not significantly different between the 2–3 and the 28–29 months old IClc (*p* = 0.61). In each box plot, dark lines represent the median of the distribution, boxes extend across the interquartile range, and whiskers extend to ±150% of the interquartile range. See list of abbreviations.

We quantified 5,882 GAD^+^ cells at 2–7 months and 6,316 GAD^+^ cells at 28–29 months to assess the effect that aging had on the density of GABAergic IC cells. As described in section “Materials and Methods,” the quantitative analysis was restricted to tissue depths that were successfully penetrated by the antibodies. Therefore, we interpret GAD^–^ cells as presumptively glutamatergic and not the result of poor GAD staining. We found similar densities of GAD^+^ cells in each subdivision at both 2–7 and 28–29 months of age (Figure 5). Our results did not find significant differences in GAD^+^ density in any of the IC subdivisions as a result of aging (all *p* ≥ 0.462; Figures 5A–C).

We examined the percentage of PN-surrounded GAD^+^ cells at 2–7 months and 28–29 months. For each subdivision at 2–7 months, we found that a minority (ICc-21.5%; ICd-16.6%; IClc = 24.7%) of PNs surround GAD^+^ cells. These percentages were not significantly different at 28–29 months (ICc-26.3%; ICd-18.3%; IClc = 26.7%) (age-by-GAD presence interaction: *p* = 0.90). We therefore interpret these data that in the FBN rat (1) at each age PNs in the IC surround non-GABAergic cells more frequently than GABAergic cells and (2) that the distribution of PN’s around GAD^+^ and GAD^–^ cells does not vary by age.

**FIGURE 6:**
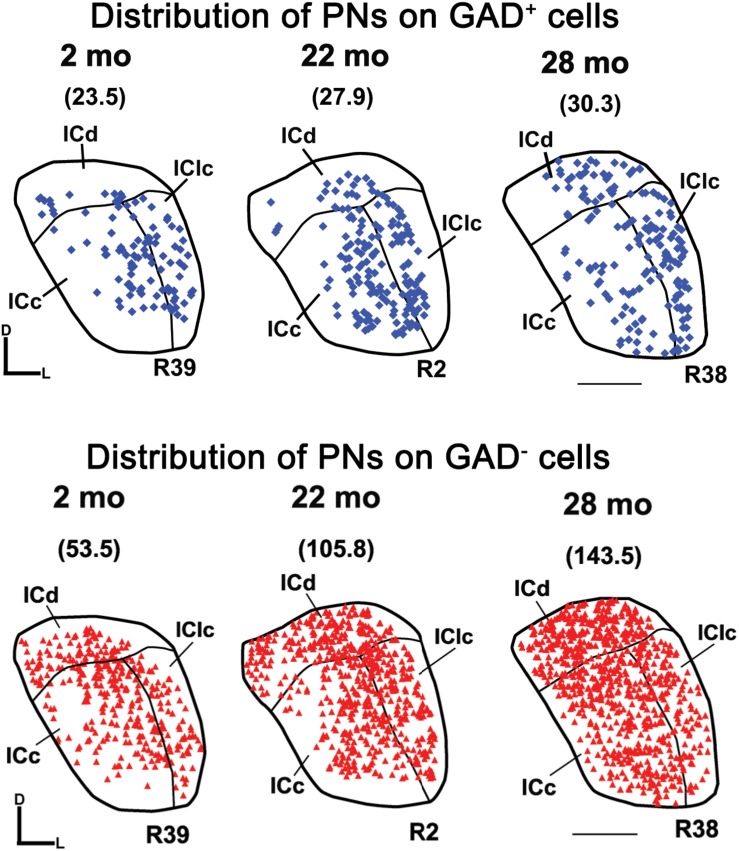
Plots showing the distribution of PNs that surround GAD^+^ cells (blue diamonds) and GAD^–^ cells (red triangles) in the IC at three age groups. Each symbol represents one PN. Densities of PNs for the individual sections are shown in parentheses. Plotted sections were chosen between interaural levels 0.12 mm – 0.48 mm; Paxinos and Watson, 1998. D, dorsal; L, lateral. Transverse sections at a mid-rostrocaudal level of the IC. Scale bar = 1 mm. See list of abbreviations.

**FIGURE 7:**
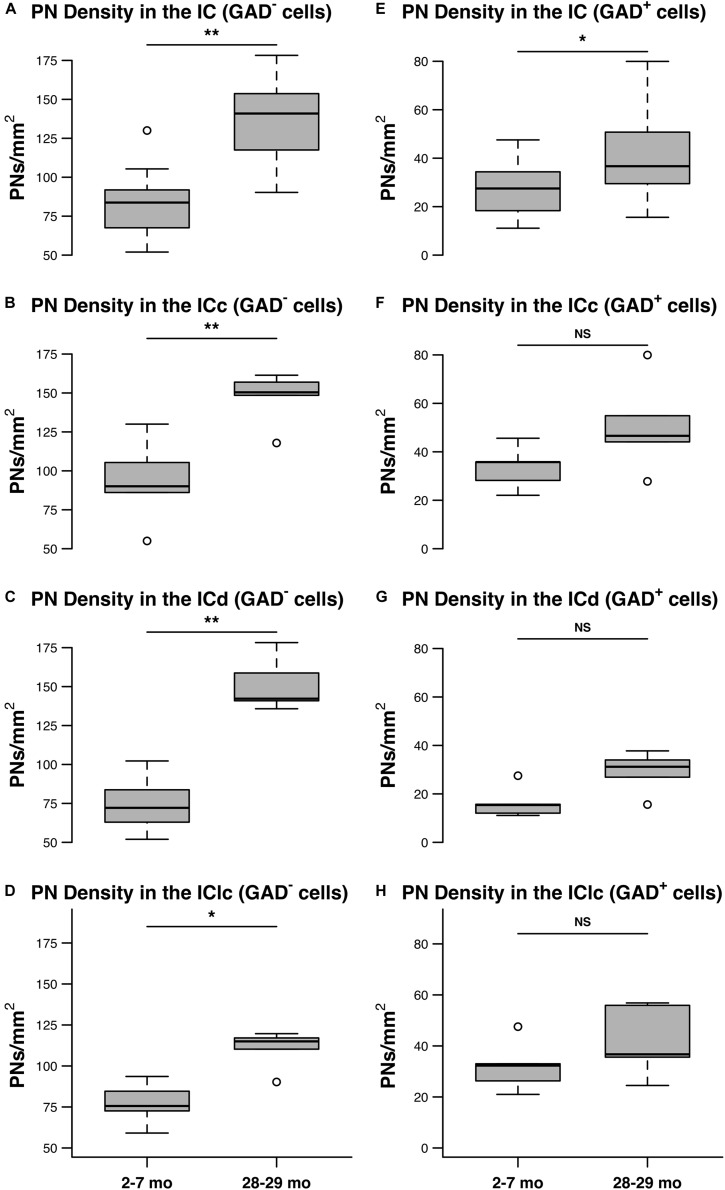
The densities of PNs increase with age on GAD^–^ and GAD^+^ cells in the IC. **(A–D)** Box plots summarizing the densities (PNs/mm^2^) surrounding GAD^–^ cells in the IC in the youngest (2–7 months) and oldest (28–29 months) age groups. **(A)** PN density significantly increased with age across the IC (**p = 0.0015). **(B)** PN density significantly increased with age in the ICc (**p = 0.0087). **(C)** PN density significantly increased in the ICd (**p = 0.0011). **(D)** PN density significantly increased in the IClc (*p = 0.046). **(E–H)** Box plots summarizing the densities (PNs/mm^2^) surrounding GAD^+^ cells in the IC in the youngest (2–7 months) and oldest (28–29 months) age groups. **(E)** PN density significantly increased with age across the IC (*p = 0.029). However, mixed effect modeling did not find a significant increase when subdivisions were considered individually **(F–H)** (ICc, *p* = 0.0723; ICd, *p* = 0.0574; IClc, *p* = 0.1140). In each box plot, dark lines represent the median of the distribution, boxes extend across the interquartile range, and whiskers extend to ±150% of the interquartile range. Circles indicate outliers beyond this range. See list of abbreviations.

Figure 6 shows the distribution of PN-surrounded GAD^+^ and PN-surrounded GAD^–^ cells across three age groups. Note that the IC sections are from the same cases and the pattern of distribution for PN-surrounded GAD^+^ cells (blue diamonds) and PN-surrounded GAD^–^ cells (red triangles) was similar (Figure 6). PN-surrounded GAD^–^ cells in the ventromedial ICc was more common in the older IC (Figure 6). In the young ICd and the IClc, PNs were more commonly found in the subdivision’s deeper layers; with age PNs were also commonly expressed in the upper layers near the tissue surface (Figure 6). We found that from the youngest group (2–7 months) to the oldest group (28–29 months) the density of PNs increased in the IC overall (*p* ≤ 0.029), regardless of GAD presences (Figure 7). Among the population of PN-surrounded GAD^–^ cells, density also significantly increased with age across all individual IC subdivisions (all *p* ≤ 0.046; Figure 7). Among the population of PN-surrounded GAD^+^ cells, mixed-effect modeling found that the increases within individual subdivisions were not significant (all *p* ≥ 0.057; Figure 7).

## Discussion

The current study examines age-related changes to the density of PNs surrounding non-GABAergic and GABAergic cells in the IC. Our main finding is that the density of PNs in the IC increased with age. When we examined the IC across three age groups, without accounting for subdivisions, there was a significant increase in the density of PNs between the 19–24 and 28–29 months age groups. As the subdivisions were examined independently we found significant increases in the density of PNs at 28–29 months in the ICc and the ICd. We further found that PNs in the FBN rat IC more commonly surround non-GABAergic cells at all ages examined and that the increase of PN density late in age occurred on both non-GABAergic and GABAergic populations. We examined the age-related increase of PN density with respect to the presence of GAD between our youngest and oldest age groups. Mixed-effect modeling indicated that PN density increased with age on GAD^–^ and GAD^+^ cells across the IC overall, ignoring distinctions among subdivisions. When distinctions among subdivisions were not ignored, the model revealed a significant increase in the density of PN-surrounded GAD^–^ cells in each subdivision. Significance was not detected among subdivisions for densities of PN-surrounded GAD^+^ cells, however, lower sample size within the mixed-effect model limited statistical power to detect significant difference within subdivisions (*p* = 0.057 – 0.114). Based on previous literature we anticipated a potential decline in the number of GAD^+^ cells present in our older tissue (Caspary et al., 1990), which could impact the interpretation of our measures. However, we found no significant age-related changes in the number or density of GAD^+^ cells between age groups in any IC subdivision. Thus, we presume our density measures of PNs surrounding non-GABAergic cells are not skewed by an age-related loss of GABAergic cells. In the following sections, we discuss technical aspects of our analysis and then consider some functional implications of the age-related upregulation of PNs in the IC.

### Technical Considerations

We employed WFA as it is largely accepted as a marker for the majority of PNs and has been used in many species (Belichenko et al., 1999; Härtig et al., 1999; Cant and Benson, 2006; Ajmo et al., 2008; Morawski et al., 2012; Foster et al., 2014; Sonntag et al., 2015; Beebe and Schofield, 2018). However, the molecular compositions of PNs may vary between brain regions and/or be differentially regulated with aging (Friauf, 2000; Brückner et al., 2003; Ajmo et al., 2008; Giamanco et al., 2010; Wang and Fawcett, 2012; Karetko-Sysa et al., 2014). Methods that use antibodies to CSPG molecules (aggrecan, brevican, phosphacan) can label populations of PNs that WFA does not reveal (Lurie et al., 1997; Ajmo et al., 2008; Karetko-Sysa et al., 2011; Blosa et al., 2013; Sonntag et al., 2015). Furthermore, immunohistochemistry of PNs depends on the state of glycosylation of the proteoglycans being detected (Matthews et al., 2002). Taken together, while the majority of PNs were likely labeled in the current study it is likely that the entire population of PNs in the IC is not reflected.

We used exclusively male FBN rats in the current study as we did not have equal representative numbers of female FBN rats. Having sufficient numbers of both sexes, especially for aging research, is necessary to make adequate conclusions of the results as many if not all rodent species have varying survival rates across sexes. In the case of the current study, as reported by the National Institute on Aging, FBN male rats have a higher percentage of survival at a given age than females. Thus, the mortality rate of an age group of males spanning 19–24 months is not equivalent to a group 19–24 months old females.

Lipofuscin, an autofluorescent pigment, appears in the cytoplasm as early as 3–4 months of age and continues to accumulate until death. To better resolve the PN staining and GAD signals, we used fluorescent tags visible in near-infrared wavelengths where the lipofuscin autofluorescence in the IC is diminished (Robinson et al., 2019).

We have found incomplete labeling when combining neuron specific antibodies such as NeuN with antibodies to GAD in the FBN rat. We therefore chose to use the fluorescent Nissl stain NeuroTrace (NT), which labels both neurons and glia, to identify GAD^–^ somas. To avoid quantifying glia we (1) excluded cells that were small and had a very small cytoplasm/nucleus ratio and (2) quantified tissue with four individuals to cross-validate our neuron numbers. It is possible that a few glia were included in our analysis, however given the large number of cells quantified in the current study a few glia cells would not have a substantial effect on our results.

There have been reports on the loss of GAD cells/expression in the aging IC (Caspary et al., 1990; Gutiérrez et al., 1994; Burianova et al., 2009). In the current study we find that the density of GAD^+^ cells is unchanged with age (Figure 5). Perhaps this reflects previous findings that GABAergic cells in the IC were not lost with age (Helfert et al., 1999; Gleich et al., 2014). While our methods for quantification and GAD immunohistochemistry demonstrate similar numbers of GABAergic cells in the young and old IC, those cells could be producing less GAD with age as Burianova et al. (2009) has carefully demonstrated. In fact, current studies in our lab (not shown) employing small molecule fluorescent *in situ* hybridization show that GABAergic cells in the ICc produce less GAD mRNA during aging. Additionally, we did not take measures such as optical density and intensity analysis to gauge the loss of GAD expression, we simply defined cells as GAD^+^ if there was somatic expression.

### Comparison to Previous Studies

Plasticity in the developing visual system is where much of our knowledge of PN function is derived from (see reviews Wlodarczyk et al., 2011; Wang and Fawcett, 2012). In the auditory system only a handful of studies have investigated the presence of PNs in the auditory brainstem, none of which examined the effect of old age on PN expression in the IC (Bertolotto et al., 1996; Hilbig et al., 2007; Foster et al., 2014; Sonntag et al., 2015; Beebe et al., 2016; Fader et al., 2016; Beebe et al., 2018; Beebe and Schofield, 2018; Heusinger et al., 2019; Mansour et al., 2019; Sucha et al., 2019). Here we used the FBN rat, a recommended model for aging studies by the National Institute on Aging and determined that density of PNs increases with age in the IC. As the current study is the first investigation of PNs in the aging IC it is difficult to draw direct comparisons of our results to the past studies listed above. However, our data reflects similar age-related increases of PNs found in the auditory thalamus, somatosensory, visual and prefrontal cortices and the dentate gyrus (Tanaka and Mizoguchi, 2009; Yamada and Jinno, 2013; Karetko-Sysa et al., 2014; Ueno et al., 2018; Quraishe et al., 2019). Additionally, Fader et al. (2016) found that the density of PNs in the IC began to increase between 3 (mature adult) and 9 (middle-age) month old mice. It has been demonstrated that CSPGs in the contralateral IC increase for a period of time after a unilateral sensory deafferentation via cochlear ablation (Heusinger et al., 2019). This may reflect the current study in that the IC’s response to cochlear insult, age or ablation, is to increase the presence of PNs. Interestingly, studies in the auditory cortex have considerable differences; Brewton et al. (2016) reported a decrease of PNs with age, while Ueno et al. (2018) reported an increase and Cisneros-Franco et al. (2018) found that PNs increased throughout life and then decreased during old age. In the current study we found that age-related increases of PNs were greater in the ICc and ICd than they were in the IClc. In several species, including mouse and rat, layer II of the developing IClc has a unique feature in that GABAergic cells and boutons form clusters termed as “GABAergic modules” (Chernock et al., 2004; Gay et al., 2018). Similar to Fader et al. (2016) we examined the IClc GABAergic modules and found that PN expression was not significantly different (not shown) than in the non-modular IClc. We conclude that PN upregulation with age is not uniform in the IC and therefore various PN-associated functions of the IC are likely to be differentially affected by age.

PNs, especially in cortex, are often associated with fast-spiking inhibitory cells (Härtig et al., 1992; Hensch, 2005; Nowicka et al., 2009). In the mammalian IC roughly a quarter of the cells are GABAergic while the remaining cells are presumptively glutamatergic (Oliver et al., 1994; Merchán et al., 2005; Mellott et al., 2014a). A major finding of the current study was that PNs most commonly surrounded non-GABAergic cells in the young IC, similar to the findings of Fader et al. (2016). Additionally, we found that the age-related increase of PNs, while occurring on both populations, was more commonly associated with non-GABAergic cells. It is well established that both GABAergic and non-GABAergic cells throughout the ICc, IClc, and ICd send ascending projections to the auditory thalamus (Peruzzi et al., 1997; Mellott et al., 2014a, b). Thus, our results suggest that age-related increase of PNs may affect the processing of numerous ascending pathways. The relationship between PNs and GABAergic cells in the IC appears to be species, and perhaps even strain specific (guinea pig, Foster et al., 2014; mouse, Fader et al., 2016; rat, current study). However, it is clear that in these studies that PNs surround both GABAergic and non-GABAergic populations of IC cells. We are not the first to report an age-related increase of PNs on excitatory circuits as Karetko-Sysa et al. (2014) demonstrated the increase of WFA labeled PNs with age occurs predominantly on non-GABAergic cells in sensory cortex. However, they also demonstrated a small but significant loss of Cat-301 labeled PNs with age in sensory cortex. Given that WFA and Cat-301 both bind to epitopes on the aggrecan molecule and likely reflect two different sugars (Matthews et al., 2002); it appears that aging can differentially regulate separate populations of PNs in cortex (Karetko-Sysa et al., 2014).

### Potential Functional Roles of PNs in Age-Related Hearing Loss

Age-related hearing loss is routinely viewed as a combination of peripheral and central processing deficits that can manifest as temporal processing disorders (see reviews Frisina, 2009; Liberman, 2017). In the IC, GABAergic inhibition has been a focus of hearing loss and aging studies as GABA is (1) essential for maintaining temporal precision and extracting a signal from noise (Faingold et al., 1989; Frisina, 2001; Walton et al., 2002) and (2) downregulated with age (see review Caspary et al., 2008; Syka, 2010). A common view is that the downregulation of GABA in the aging IC may be in response to age-related cochlear deafferentation which leads to reduced excitation in the central auditory system (Caspary et al., 2008; Auerbach et al., 2014; Parthasarathy et al., 2014, 2018; Parthasarathy and Kujawa, 2018). This potential compensatory mechanism may maintain the correct balance of excitation and inhibition needed for temporal processing as one ages. However, IC cells lose their precise temporal locking when GABAergic processing is interrupted; thus, temporal precision in the aged IC is often disrupted with the age-related loss of GABA (Le Beau et al., 1996; Palombi and Caspary, 1996a; Frisina, 2001; Walton et al., 2002; Parthasarathy and Bartlett, 2011; Parthasarathy et al., 2018).

The current study demonstrates a significant increase of PNs, predominantly surrounding non-GABAergic cells, in the IC at 28–29 months. Interestingly, in the FBN rat, hearing thresholds at specific frequencies increase at 24 months and continue to increase across numerous frequencies at 28 months (Cai et al., 2018). It appears that the age-related increase of PNs in the IC may be beginning during the same window of time when age-related increases of hearing thresholds occur. Whether age-related peripheral deficits such as cochlear deafferentation leading to elevated hearing thresholds bring about changes in the central auditory system via increased PN expression has not been explored. PNs are known to serve many functions in the central nervous system. We discuss below several of these functions and why an increase of PNs in the IC may be relevant to age-related hearing loss.

PNs in the central nervous system are most commonly associated with reduced structural synaptic plasticity (Corvetti and Rossi, 2005; Frischknecht et al., 2009; Karetko-Sysa et al., 2014; Sonntag et al., 2015; see review: Duncan et al., 2019). The role of PNs in structural synaptic plasticity has been further confirmed with many studies demonstrating that the removal of PNs in cortex, via enzymatic digestion, leads to increased plasticity in the visual cortex, learning and memory circuits and fear conditioning (Pizzorusso et al., 2006; see reviews: Wang and Fawcett, 2012; Duncan et al., 2019; Testa et al., 2019). In the current study we demonstrate a significant increase of PNs on both non-GABAergic and GABAergic IC cells. The additional PNs from aging are likely creating a less plastic environment for those IC circuits they surround. It would be logical to assume that IC cells with reduced structural synaptic plasticity are less likely to appropriately respond to the continual age-related loss of GABA. Thus, as the loss of plasticity reaches an eventual threshold, inhibition in the IC may be altered as given cell populations are no longer able to balance the incoming inhibitory and excitatory inputs. These changes to the inhibition in the IC may reflect the disrupted temporal processing that occurs in aged populations (Palombi and Caspary, 1996b; Walton et al., 1998, 2002; Frisina, 2001; Parthasarathy and Bartlett, 2011). On the other hand, the increase of PNs may serve to stabilize existing circuits that are performing well by maintaining the correct excitatory-inhibitory balance throughout life (Karetko-Sysa et al., 2014). Perhaps the IC is upregulating PNs late in life to stabilize and protect circuits that have maintained their temporal precision throughout aging.

PNs can regulate and promote functional synaptic plasticity (Bukalo et al., 2001; Balmer et al., 2009; de Vivo et al., 2013; Blosa et al., 2015; Bosiacki et al., 2019). Interestingly, a recent study has shown that PN expression in the medial subdivisions of the auditory thalamus may be responsible for precise neural firing in the aging brain (Quraishe et al., 2019). In the IC, both GABAergic and glutamatergic IC cells (1) receive GABAergic input from numerous subcortical sources and (2) change the subunit composition of their postsynaptic GABA_A_ receptors in a non-linear fashion early and throughout life (Milbrandt et al., 1996, 1997; Caspary et al., 1999; Kelly and Caspary, 2005; Robinson et al., 2019). In the rat it has been demonstrated that excitatory and inhibitory synapses in the ICc undergo rearrangement by 19 and 28 months and are each reduced by ∼25–30% (Helfert et al., 1999). Taken together, we assume that the IC is compensating for the age-related changes to GABA_A_ receptors and the decline of synapses rather well for most of life, as deficits to temporal and speech processing are typically present only in aged listeners (Pichora-Fuller and Souza, 2003; Walton, 2010; Ruggles et al., 2011; Füllgrabe et al., 2015). As noted above, there have been many studies on the presence of PNs in the central auditory system. However, the specific roles that PNs serve in the auditory system, let alone the aging auditory system, are still largely unknown (see review Sonntag et al., 2015). Given the numerous functions to synaptic transmission that PNs have been known to contribute (see reviews Sonntag et al., 2015; Testa et al., 2019), we can make one of many conjectures regarding the relevance of PNs to the aging IC. Perhaps the presence of PNs in the IC until old age may be necessary to promote functional synaptic plasticity as receptors change their subunit composition and stabilize GABAergic synapses to maintain correct levels of inhibition as cochlear excitation is gradually lost. It is possible that the significant increase of PNs in the current study is a homeostatic mechanism to compensate for a critical loss of synapses in the aging IC. The PNs may be attempting to maintain an overall synaptic integrity and “lock” remaining GABAergic synapses in place. However, whether the upregulation of PNs is indeed a homeostatic mechanism the routine onset of hearing deficits in old age demonstrate that such compensation is likely insufficient. On the other hand, the presence of additional PNs in old age may not be related to the auditory processing that occurs in the IC.

There are numerous studies that have investigated the link between aging and increases in oxidative stress (see review Liguori et al., 2019). PNs are also known to protect neurons against oxidative stress (Morawski et al., 2004; Suttkus et al., 2012, 2014; Cabungcal et al., 2013). Enzymatic degradation studies have demonstrated that PNs protect fast-spiking parvalbumin cells in cortex (Cabungcal et al., 2013). Specifically, it has been shown that PN with the CSPG aggrecan can protect neurons from iron induced oxidative stress (Suttkus et al., 2014). The proposed mechanism is that PNs scavenge and bind redoxactive iron, thus reducing the oxidative potential in the cell’s microenvironment (Suttkus et al., 2014). It is also known that many PNs in the rat IC contain aggrecan (Beebe and Schofield, 2018). Collectively, the previous literature raises a critical point in respect to aging in that to understand the function of PNs in the aged IC it will be important to determine whether the upregulated PNs have varying molecular compositions. Understanding the molecular composition of the upregulated PNs may shed light on whether the upregulated PNs in the IC may protect neurons from oxidative stress. It is logical to assume that the increase in PNs we observe in the aging IC may be in direct response to age-related increases of oxidative stress and not to a feature of auditory processing or the age-related loss of GABA. The potential reduced structural plasticity and disruptions to auditory processing in old age that may accompany the increase of PNs may be a “lesser of evils” as PNs and their CSPGs attempt to protect IC cells from cytotoxicity and cell death. Whether or not the increase of PN density is resultant to any one specific biological event, the processing of both non-GABAergic and GABAergic cells in the IC are likely affected by changes in structural and synaptic plasticity at old age. The age-related loss of GABAergic synapses to both non-GABAergic and GABAergic cells in the ICc (Helfert et al., 1999), combined with the increase of PN density on both cell populations, may create an environment in that inhibitory processing is placed out of balance with excitatory processing. Furthermore, the IC cells with PNs may not be able to adapt to the reductions in GABA as reduced plasticity occurs. Taken together, the presence of additional PNs during old age may disrupt temporal processing in the IC reliant on the precise balance of GABAergic and non-GABAergic inputs and ultimately may lead to features of age-related hearing loss.

Although there are numerous cellular functions assigned to PNs in the central nervous system, how PNs affect the processing of auditory signals in the IC and may contribute to age-related hearing loss remains to be determined. The current study suggests that the age-related upregulation of PNs is largely targeting non-GABAergic, and to a lesser extent GABAergic, cells in the IC. However, future studies are required to determine how PNs affect the processing of excitatory and inhibitory IC circuits. Determining whether PNs that are upregulated during old age are of different molecular compositions than “young” PNs and/or are surrounding cells of specific IC pathways, and/or targeting synapses in the IC from specific nuclei will be key in understanding the functions of PNs in the aged IC.

## Conclusion

The present study demonstrates that PNs are upregulated with age in the IC of the FBN rat. Specifically the increase was observed late in life. The greatest increases of PNs throughout the IC occurred in the ICc and the ICd. The upregulation of PNs also occurred on both non-GABAergic and GABAergic IC cells. Thus, the processing of numerous output pathways of the IC are likely affected by the increase PNs during old age. The current study found that PNs in the FBN rat IC more commonly surrounded GAD^–^ cells at all ages examined. The percentage of PNs surrounding GAD^+^ cells at young ages was not significantly different than it was at older ages. Thus, the distribution of the upregulated PNs with age largely does not vary between the two populations of cells examined. Mixed-effect modeling did find that the increase of PNs surrounding non-GABAergic and GABAergic cells across the IC at 28–29 months was significant from 2 to 3 months. Interestingly, the increase of PNs in the IC occurs at ages often associated with age-related hearing loss. Further investigations will be needed to determine why PNs in the IC are upregulated with age and whether they play unique roles at specific ages.

## Data Availability Statement

All datasets generated for this study are included in the article/supplementary material.

## Ethics Statement

The animal study was reviewed and approved by the Northeast Ohio Medical School IACUC.

## Author Contributions

JM, AM, LH, and MR generated the data. JY and AM performed the data analysis. AM and JM designed the experiments and wrote the manuscript.

## Conflict of Interest

The authors declare that the research was conducted in the absence of any commercial or financial relationships that could be construed as a potential conflict of interest.

## Abbreviations

CSPG: chondroitin sulfate proteoglycans
ECM: extracellular matrix
FBN: Fischer Brown Norway
GABA: gamma-aminobutyric acid
GAD: glutamic acid decarboxylase
GAD^–^: GAD-immunonegative
GAD^+^: GAD-immunopositive
IC: inferior colliculus
ICc: central nucleus of the inferior colliculus
ICd: dorsal cortex of the inferior colliculus
IClc: lateral cortex of the inferior colliculus
NT: NeuroTrace
PNs: perineuronal nets
WFA: *Wisteria floribunda* agglutinin.
